# Exploiting likely-positive and unlabeled data to improve the identification of protein-protein interaction articles

**DOI:** 10.1186/1471-2105-9-S1-S3

**Published:** 2008-02-13

**Authors:** Richard Tzong-Han Tsai, Hsi-Chuan Hung, Hong-Jie Dai, Yi-Wen Lin, Wen-Lian Hsu

**Affiliations:** 1 Department of Computer Science and Engineering, Yuan Ze University, Chung-Li, Taoyuan 32003, Taiwan, R.O.C; 2 Institute of Information Science, Academia Sinica, Nankang, Taipei 115, Taiwan, R.O.C

## Abstract

**Background:**

Experimentally verified protein-protein interactions (PPI) cannot be easily retrieved by researchers unless they are stored in PPI databases. The curation of such databases can be made faster by ranking newly-published articles' relevance to PPI, a task which we approach here by designing a machine-learning-based PPI classifier. All classifiers require labeled data, and the more labeled data available, the more reliable they become. Although many PPI databases with large numbers of labeled articles are available, incorporating these databases into the base training data may actually reduce classification performance since the supplementary databases may not annotate exactly the same PPI types as the base training data. Our first goal in this paper is to find a method of selecting likely positive data from such supplementary databases. Only extracting likely positive data, however, will bias the classification model unless sufficient negative data is also added. Unfortunately, negative data is very hard to obtain because there are no resources that compile such information. Therefore, our second aim is to select such negative data from unlabeled PubMed data. Thirdly, we explore how to exploit these likely positive and negative data. And lastly, we look at the somewhat unrelated question of which term-weighting scheme is most effective for identifying PPI-related articles.

**Results:**

To evaluate the performance of our PPI text classifier, we conducted experiments based on the BioCreAtIvE-II IAS dataset. Our results show that adding likely-labeled data generally increases AUC by 3~6%, indicating better ranking ability. Our experiments also show that our newly-proposed term-weighting scheme has the highest AUC among all common weighting schemes. Our final model achieves an F-measure and AUC 2.9% and 5.0% higher than those of the top-ranking system in the IAS challenge.

**Conclusion:**

Our experiments demonstrate the effectiveness of integrating unlabeled and likely labeled data to augment a PPI text classification system. Our mixed model is suitable for ranking purposes whereas our hierarchical model is better for filtering. In addition, our results indicate that supervised weighting schemes outperform unsupervised ones. Our newly-proposed weighting scheme, TFBRF, which considers documents that do not contain the target word, avoids some of the biases found in traditional weighting schemes. Our experiment results show TFBRF to be the most effective among several other top weighting schemes.

## Background

Most biological processes, including metabolism and signal transduction, involve large numbers of proteins and are usually regulated through protein-protein interactions (PPI). It is therefore important to understand not only the functional roles of the involved individual proteins but also the overall organization of each biological process [1].

Several experimental methods can be employed to determine whether a protein interacts with another protein. Experimental results are published and then stored in protein-protein interaction databases such as BIND [2] and DIP [3]. These PPI databases are now essential for biologists to design their experiments or verify their results since they provide a global and systematic view of the large and complex interaction networks in various organisms.

Initially, the results were mainly verified and added to the databases manually. Since 1990, the development of large-scale and high-throughput experimental technologies such as immunoprecipitation and the yeast two-hybrid model has boosted the output of new experimental PPI data exponentially [4]. It becomes impossible to perform the relying curation task on the formidable number of existing and emerging publications if it relies solely on human effort. Therefore, information retrieval and extraction tools are being developed to help curators. These tools should be able to examine enormous volumes of unstructured texts to extract potential PPI information. They usually adopt one of two general approaches: (1) extracting PPI information directly from the literature [5-9]; (2) finding articles relevant to PPI first, and then extracting the relevant information from them.

The second approach is more efficient than the first. It extracts fewer false positive PPIs because the total number of biomedical articles is very large and most of them are not directly relevant to PPI. Therefore, in this paper, we focus on the first step of the second approach: finding articles relevant to PPI.

Most methods in this approach formulate the article-finding step as a text classification (TC) task, in which articles relevant to PPI are denoted as positive instances while irrelevant ones are denoted negative. We refer to this task as the PPI-TC task from now on. One advantage of this formulation is that the methods commonly used in general TC systems can be modified and applied to the problem of identifying PPI-relevant articles.

In general TC tasks, machine-learning approaches are state-of-the-art. Support vector machines [10] or Bayesian approaches [11] are two popular examples. These approaches can achieve very high accuracy but they also require a sufficient number of training data, including both positive and negative instances.

**Figure 1:**
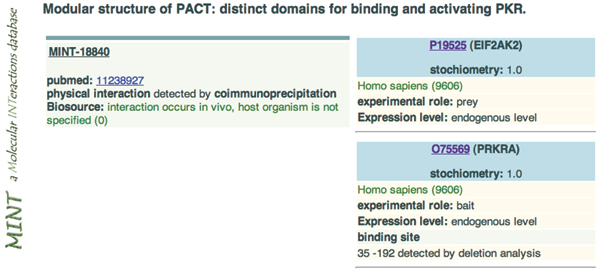
A PPI record in the MINT database.

In PPI-TC, the definition of 'PPI-relevant' varies with the database for which we curate. Most PPI databases define their standard according to Gene Ontology, a taxonomy that classifies all kinds of protein-protein interactions. Each PPI database may only annotate a subset of PPI types; therefore, only some of these types will overlap with a different PPI database. In PPI databases, each existing PPI record is associated with its literature source (PMID). Figure 1 shows a PPI record of the MINT [12] database. It shows that the article with PubMed ID:11238927 contains information about the interaction between P19525 and O75569, where P19525 and O75569 are the primary accession numbers of two proteins in the UniProt database. These articles can be treated as PPI-relevant and as true positive data. However, to employ mainstream machine-learning algorithms and improve their efficacy in PPI-TC, there are still two major challenges. The first is how to exploit the articles recorded in other PPI databases. Since other databases may partially annotate the same PPI types as the target database, articles recorded in them can be treated as likely-positive data. If more effective training data are included, the feature space will be enlarged and the number of unseen dimensions reduced. Considering these articles may increase the generality of the original model. The second challenge is a consequence of the first: To use likely-positive data we must collect corresponding likely-negative data or the ratio of positive to negative data will become unbalanced.

In this paper, our primary goal is to develop a method for the selection and exploitation of likely-positive and likely-negative data. In addition, since term-weighting is an important issue in general TC tasks and usually depends on the corpus and domain, we also investigate the secondary issue of which scheme is best suited to PPI-TC. PPI-TC systems have two possible uses for database curators. One is merely as filters to remove irrelevant articles. The other is to rank articles according to their relevance to PPI. We will first describe our experience of building our PPI-TC system in the "System overview" section. We will then use different evaluation metrics to measure system performance and discuss different configurations in the remaining sections.

## System overview

**Figure 2:**
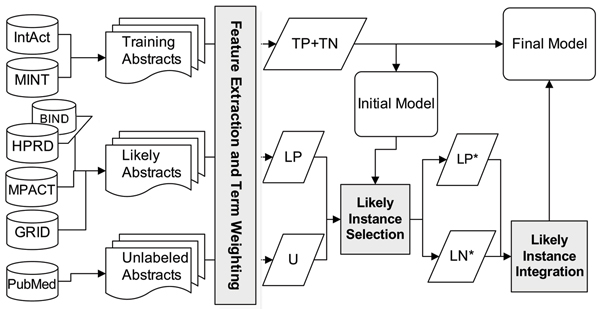
An overview of our protein-protein interaction text classification system.

Figure 2 shows an overview of our PPI-TC system. This system comprises the following components; those shown as boldface in the figure are the aims of this paper:

### Step 1: Dataset preparation

We use the training (true positive and true negative; annotated 'TP+TN' in Figure 2) and likely positive ('LP' in Figure 2) datasets from BioCreAtIvE-II interaction abstract subtask [13] and the unlabeled datasets ('U' in Figure 2) from PubMed. The treatment applied on LP and U will be described in Step3. The preparation of these datasets is detailed in the Datasets subsection of the Methods section. The size of each dataset is shown in Table 1.

**Table 1:** Datasets used in our experiment

	Dataset	Size (# of abstracts)
Training	True positive (TP)	3,536
	True negative (TN)	1,959
	Likely-positive (LP)	18,930
	Unlabeled (U)	105,000
Test	Positive	338
	Negative	339

Their source databases are depicted in Figure 2. For each abstract, we remove all punctuation marks, numbers and stop words in the pre-processing step.

### Step 2: Feature extraction and term weighting

The most typical feature representation in TC systems is bag-of-word (BoW) features, in which a term in document is converted into a feature vector. This feature vector is calculated by a term-weighting function. Then the classification of these feature vectors can be modeled with existing classifiers such as support vector machines (SVM).

It is very important for SVM-based TC to select a suitable term-weighting function to construct the feature vector because SVM models are sensitive to the data scale, i.e. they are dominated by some very wide dimensions. A feasible term-weighting function emphasizes informative or discriminating words by allowing their feature values to occupy a larger range, increasing their influence in the statistical model. In addition to the simplest binary feature, which only indicates the existence of a word in a document, there are currently numerous term-weighting schemes that utilize term frequency (TF), inverse document frequency (IDF) or statistical metrics information. Lan et al. [14] pointed out that the popularly-used TF-IDF method has not performed uniformly well with respect to different data corpora. The traditional IDF factor and its variants were introduced to improve the discriminating power of terms in the traditional information-retrieval field. However, in text categorization, this may not be the case. Hence, they proposed a new supervised weighting scheme, TFRF, to improve the term's discriminating power. Another popular supervised weighting scheme BM25 [15] has been shown to be efficient in recent studies and tasks on IR [16]. We have not seen any previous attempt to apply BM25 to TC, perhaps because it was originally designed for applications with input query, such as searching or question answering.

Inspired by the idea of Lan et al. and by BM25, we propose a new supervised weighting scheme, TFBRF, which avoids some biases in PPI-TC problem. The details of TFBRF will be illustrated in the "Methods" section. We will compare it with other popular general-TC term weighting schemes mentioned above in "Result" section.

### Step 3: Selecting likely-positive and negative data

The base training set (from BioCreAtIvE-II IAS) contains only limited numbers of TP and TN data. To increase the generality of the classification model, more external resources should be introduced, such as the LP provided by BioCreAtIvE-II and external unlabelled dataset proposed by this work. For likely positive dataset, one important resource is other PPI databases; abundant PPI articles are recorded in various such databases. However, most of them only annotate a selection of all the PPI types defined in Gene Ontology. Therefore, some annotations may match the criteria of the target PPI database while others may not. This means that abstracts annotated in that database can only be treated as likely-positive examples, some of which may need to be filtered out.

Another problem is that there are no negative data or even likely-negative data in any curation. Because most machine-learning-based classifiers tend to explicitly or implicitly record the prior distribution of positive/negative labels in the training data, we will obtain a model with a bias toward positive prediction if only those instances in the PPI databases are used. An imbalance in training data can cause serious problems. However, a large proportion of the biomedical literature is negative, which is exactly the opposite. More likely-negative (LN) instances should be incorporated to balance the training data, and this can be carried out in a manner similar to filtering out LP instances. Here, we introduce the external unlabelled dataset to deal with this problem.

Since there may be noisy examples in the LP and unlabeled data, we have to select reliable instances from them in order to use these data to augment our classifier. The detailed filtration is described in the "Method" section. We list the selected instances including 'selected likely positive' and 'selected likely negative' instances in Table 2.

**Table 2:** The selected likely datasets

Dataset	Size (# of abstracts)
Selected Likely-positive (LP*)	8862
Selected Likely-negative (LN*)	10000

### Step 4: Exploiting likely-positive and negative data

The next step is to integrate the selected likely data into the training set to build the final model. Here, we employ and compare two integration strategies: 1) directly mixing the selected likely data with the original training data, called a 'mixed model'; or 2) building an ancillary model with these likely data and encoding their prediction as features in the final model, called a 'hierarchical model'. The details of these two strategies can be found in the "Methods" section.

## Evaluation metrics

In this paper, we employ the official evaluation metrics of BioCreAtIvE II, which assess not only the accuracy of classification but also the quality of ranking of relevant abstracts.

### Evaluation metrics for classification

The classification metrics examine the prediction outcome from the perspective of binary classification. The value terms used in the following formulas are defined as follows: True Positive (TP) represents the number of correctly classified relevant instances, False Positive (FP) the number of incorrectly classified irrelevant instances, True Negative (TN) the number of correctly classified irrelevant instances, and finally, False Negative (FN) the number of incorrectly classified relevant instances.

The classification metrics used in our experiments were precision, recall and F-measure. The F-measure is a harmonic average of precision and recall. These three metrics are defined as follows:

$$\begin{array}{c} \begin{array}{cc} Precision=\frac{TP}{TP+FN}, & Recall=\frac{TP}{TP+FN} \end{array}\\ \text{F-measure}=\frac{2\cdot Precision\cdot Recall}{Precision+Recall} \end{array}$$

### Evaluation metrics for ranking

Curation of PPI databases requires a classifier to output a ranked list of all testing instances based on the likelihood that they will be in the positive class, as opposed to only a binary decision. The curators can then either specify a cutoff to filter out some articles on the basis of their experience, or give higher priority to more highly ranked instances.

The ranking metric used in our experiments is AUC, the area under the receiver operating characteristic curve (ROC curve). The ROC curve is a graph of the fraction of true positives (TPR, true positive rate) vs. the fraction of false positives (FPR, false positive rate) for a classification system given various cutoffs for output likelihoods, where

$$\begin{array}{cc} TPR=\frac{TP}{TP+FN}, & FPR=\frac{FP}{FP+TN} \end{array}$$

When the cutoff is lowered, more instances are considered positive. Hence, both TPR and FPR are increased since their numerators become larger but their denominator, denoting the total number of positive instances, remains constant. The more positive instances are ranked above the negative ones by the classification system, the faster TPR grows in relation to FPR as the cutoff descends. Consequently, higher AUC values indicate more reliable ranking results.

### Difference between F-Measure and AUC

F-Measure measures a classifier's best classification performance. On the other hand, AUC measures the probability of a threshold classifier that it rates a randomly chosen positive sample higher than a randomly chosen negative sample. [17,18] AUC is more suitable for applications that require ranking as it provides a measure of classifier performance that is independent of a cutoff threshold. Therefore, F-Measure tends to measure the classifier's performance on a specific threshold while AUC tends to measure a classifier's overall ranking ability. The importance of F-Measure and AUC depends on the application. For filtering, F-Measure is more important. For ranking, AUC is more suitable.

## Results

### Exploiting likely-positive and negative data

In this section, we examine the performance improvement brought by exploiting unlabeled and likely labeled data. We use the initial model, which is only trained on TP+TN data (see Figure 2), as the baseline configuration. To exploit unlabeled data and likely labeled data, we construct two different models – the mixed model and the hierarchical model. The construction procedures of these two models are detailed in the "Methods" section.

**Figure 3:**
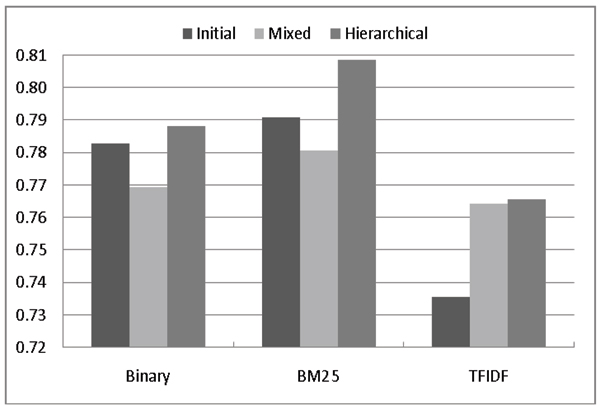
Impact of adding likely data on different term weighting schemes (F-measure).

**Figure 4:**
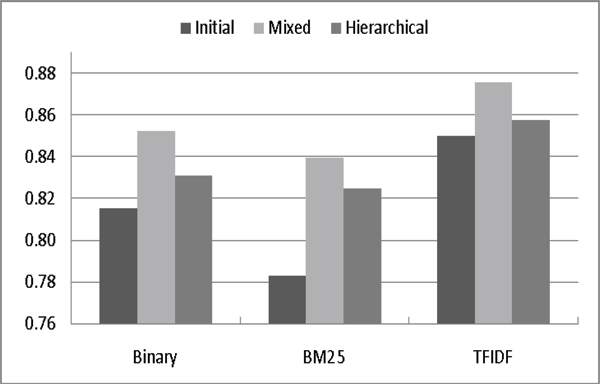
Impact of adding likely data on different term weighting schemes (AUC).

Figures 3 and 4 compares the F-Measures and AUC scores of the three models. In order to focus on a comparison of how to exploit likely-positive and negative data, we only use the most common weighting schemes: Binary, BM25 and TFIDF. These figures show that irrespective of the weighting scheme used, the hierarchical model generally has higher F-measures while the mixed model has higher AUCs. Also, regardless the weighting scheme, the initial model always has the worst AUC value, meaning that its ranking quality is also the worst. These results suggest that exploiting LP*+LN* data can refine the ranking quality effectively, which is critical for database curation.

### Employing variant term weighing schemes

**Figure 5:**
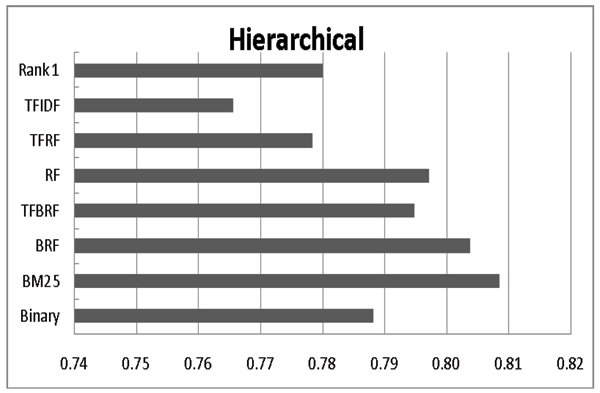
Impact of applying different term weighting schemes (F-measure). The rank 1 setting denotes the highest F-measure among all participants in BioCreAtIvE-II IAS.

**Figure 6:**
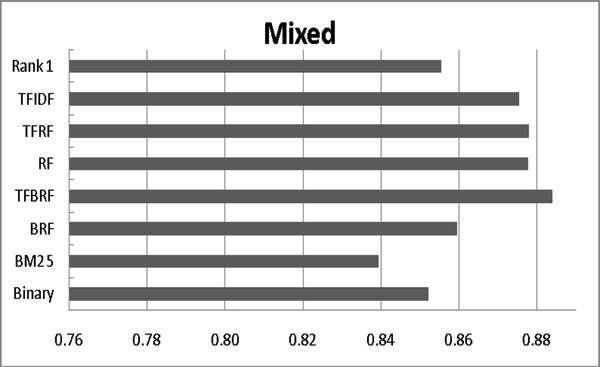
Impact of adding likely data on different term weighting schemes (AUC). The rank 1 setting denotes the highest AUC among all participants in BioCreAtIvE-II IAS.

In this section, we demonstrate the efficacy of the BM25 weighting scheme by comparing it with others. We also compare it with BioCreAtIvE's rank 1 system[13]. As shown in Figure 5, BM25 outperforms other weighting schemes in terms of F-measure within the hierarchical model. However, in terms of AUC (see Figure 6), TFBRF generally performs best. Therefore, we can conclude that if the classification model only serves as a filter, the hierarchical model with BM25 is the best choice. However, to be used as an assistant tool to help database curators, the mixed model with TFBRF is most appropriate.

Another notable result is that TFIDF, which is considered an effective term-weighting scheme in many TC and IR systems [19,20] does not significantly outperform others in this PPI-TC task. This is not surprising. There are many infrequent terms in the biomedical literature such as the names of chemical compounds, species and some proteins. These proper nouns appear rarely in publications, which gives them undue emphasis in the TFIDF weighting. However, these proper nouns, especially non-protein names, are not directly related to PPI, raising the risk of over-fitting.

## Discussion

### TFRF vs. TFBRF

Traditional term weighting schemes such as TFRF ignore term frequencies other than target terms in positive or negative documents and emphasize terms that are more frequent in the positive than the negative documents because of their hypothesis that those ignored terms are always much greater; that is, the proportion of positive instances in the training set is very small. However, this is not the case in our PPI-TC problem. We have a large number of reliable and likely positive training instances, and a nearly equivalent number of negative instances. Hence, we create a new weighting function that considers all four values. This new function is called *balanced relative frequency *(BRF) because it is similar to the relative frequency (RF) of Lan et al. In our formula, BRF takes into account the number of documents that do not contain the target word while RF does not. Detailed formulas are described in the "Method" section.

### Mixed vs. hierarchical models

As we described in the previous section, mixed models are suitable for ranking purposes whereas hierarchical models are better for filtering. Here, we discuss the reason why these two models have divergent behaviors.

For the SVMs of linear kernels, the hierarchical model is indeed equivalent to finding two separating hyperplanes:

$$\begin{array}{c} \begin{array}{c} y=w^{\prime }\cdot x \end{array}\\ y=w_{0}\cdot w^{\prime }\cdot x+w_{1}\cdot x=(w_{0}\cdot w^{\prime }+w_{1})\cdot x \end{array}$$

such that the criteria of the SVMs are optimized, where the former is trained with LP* and LN* and the latter is trained with TP and TN. Notice that the notions of the intercepts can be simplified by merging the term *b *into the weight vector ***w ***and appending a constant, say -1, to the feature vector ***x***. We can see that the strategy of using the ancillary model's output as an additional feature is an effective way to increase its influence.

Unlike in the hierarchical model, in the mixed model, all instances, whether from the true datasets or the noisy ones, are mixed together to train a separating hyperplane. In other words, the training errors on the noisy datasets are taken into consideration, so the hyperplane is more robust than that of the hierarchical model, leading to higher overall ranking ability. However, its F-measure is lower due a bias for positive data, which results from the asymmetry in the filtration thresholds applied in selecting likely negative and positive instances.

## Conclusion

The main purpose of this paper is to find a useful strategy for integrating likely positive data from multiple PPI databases with likely negative data from unlabeled sources. Our secondary intent is to compare term-weighing schemes and select that most suitable for converting documents into feature vectors. Both these issues are essential for constructing an effective PPI text classifier, which is crucial for curating databases because a good ranking can effectively reduce the total number of articles that should be reviewed given the same number of relevant articles curated.

In targeting an annotation standard of a specific PPI database, all other resources can be regarded as likely-positive. In this case, the complicated dataset integration problem can be converted into an easy filtration. Also, we can extract abundant likely-negative instances from unlimited unlabeled data to balance the training data. We demonstrate that the mixed model is suitable for ranking purposes whereas the hierarchical model is appropriate for filtering.

Different term-weighting schemes can have very different impacts on the same text classification algorithm. Being aware of the potential weakness of unsupervised term-weighting schemes such as TFIDF, we turn to some popular supervised weighting schemes and derived a novel one, TFBRF. The experimental results suggest that TFBRF and its predecessor, BM25, are favorable for ranking and filtering, respectively. This may be because they consider not only the frequencies and class labels of the documents containing the target word, but also those documents that do not contain it.

With these two strategies, our system has higher F-score and AUC than the rank 1 system of these metrics in the BioCreAtIvE-II IAS challenge, which suggests that our system can serve as an efficient preprocessing tool for curating modern PPI databases.

## Methods

In the following sections, we first introduce the machine-learning model used in our system: support vector machines. Secondly, we illustrate all the weighting schemes used in our experiments. Thirdly, we describe how our system filters out ineffective likely-positive data and selects effective likely-negative data from unlabeled data. Finally, we explain how we exploit the selected likely-positive and negative data.

### Support vector machines

The support vector machine (SVM) model is one of the best known ML models that can handle sparse high dimension data, which has been proved useful for text classification [20]. It tries to find a maximal-margin separating hyperplane <**w**, *ϕ*(**x**)> - *b *= 0 to separate the training instances, i.e.,

$$\begin{array}{c} \begin{array}{cc} \min||w|{|}^{2}+C\sum_{i}\xi^{\left(i\right)} & subject\;to \end{array}\\ \begin{array}{cc} y^{\left(i\right)}(<w,\varphi (x^{\left(i\right)})>-b)\geq 1-\xi^{\left(i\right)}, & \forall i \end{array} \end{array}$$

where **x**^(*i*) ^is the *i*th training instance which is mapped into a high-dimension space by *ϕ*(·), *y*_*i *_∈ {1, -1} is its label, *ξ*^(*i*) ^denotes its training error, and *C *is the cost factor (penalty of the misclassified data). The mapping function *ϕ*(·) and the cost factor *C *are the main parameters of a SVM model.

When classifying an instance **x**, the decision function f(**x**) indicates that **x **is "above" or "below" the hyperplane. [21] shows that the f(**x**) can be converted into an equivalent dual form which can be more easily computed:

$$\begin{array}{c} \begin{array}{c} primal\;form:f(x)=sign(<w,\varphi (x)>-b) \end{array}\\ dual\;form:f(x)=sign(\sum_{i}\alpha^{\left(i\right)}y^{\left(i\right)}K(x^{\left(i\right)},x)-b) \end{array}$$

where K(**x**^(*i*)^, **x**) = <*ϕ*(**x**^(*i*)^), *ϕ*(**x**)> is the kernel function and *α*^(*i*) ^can be thought of as *w*'s transformation.

In our experiment, we choose the following linear kernel according to our preliminary experiment results:

K(**x**^(*i*)^, **x**^(*j*)^) = <**x**^(*i*)^, **x**^(*j*)^>

Which is equivalent to

*ϕ*(**x**^(*i*)^) = **x**^(*i*)^

Finally, the cost factor *C *is chosen to be 1, which is fairly suitable for most problems.

### Term weighting

In the BoW feature representation, a document *d *is usually represented as a term vector ***v***, in which each dimension *v*_*i *_corresponds to a term *t*_*i*_. *v*_*i *_is calculated by a term-weighting function, which is very important for SVM-based TC because SVM models are sensitive to the data scale

In Table 3, we list the symbols representing the number of positive and negative documents that contain and do not contain term *t*_*i*_.

**Table 3:** The contingency table for *document frequency *of term *t*_*i *_in different classes. ¬*t*_*i *_stands for all words other than *t*_*i*_

Class	*t_*i*_*	¬*t_*i*_*
Positive	*w*	*x*
Negative	*y*	*z*

With this table, we defined usually term weighting schemes as follows:

Binary(ti,d)={1,if ti ∈ d0,otherwiseTFd(ti)=ti's term frequency in d|d|TFIDF(ti,d)=TFd(ti)⋅log⁡w+x+y+zw+y, andTFRF(ti,d)=TFd(ti)⋅log⁡(2+wy)BM25(ti,d)=2QF(ti)QF(ti)+1⋅TFd(ti)(Ld+1)+2TFd(ti)⋅log⁡(wy⋅xz)

BM25 [15] is a popular supervised weighting scheme which has been shown to be efficient in recent studies and tasks on IR. We adopt it to TC due to it was originally designed for applications with input query, such as searching or question answering, For BM25, in this paper, the query frequency QF(·) is always set to 1, so the first term in the equation is canceled. The main reason we are interested in this scheme is its last term, log((*w*/*y*)·(*x*/*z*)), which places no emphasis on either positive or negative classes but exploits class label information to examine the discriminating power of *t*_*i*_. Another characteristic of BM25 is its second term, which (relative to other schemes) de-emphasizes the frequency of *t*_*i*_.

In addition to above weighting schemes, we propose a new supervised weighting scheme, TFBRF, as follows:

$$\begin{array}{c} \begin{array}{c} BRF(t_{i},d)=\log\left(\frac{w}{y}\cdot \frac{x}{z}\right)\\ TFBRF(t_{i},d)=TF_{d}(t_{i})\cdot BRF(t_{i},d)=TF_{d}(t_{i})\cdot \log\left(\frac{w}{y}\cdot \frac{x}{z}\right) \end{array} \end{array}$$

### Datasets

The protein interaction article subtask (IAS) in BioCreAtIvE II [13] is the most important benchmark for PPI-TC. The training set comprises three parts: true positive (TP), true negative (TN) and likely-positive (LP), as shown in Table 1. The TP (PPI-relevant) data were derived from the content of the IntAct [22] and MINT [12] databases, which are not organism-specific. TN data were also provided by MINT and IntAct database curators. The LP data comprise a collection of PubMed identifiers of articles that have been used to annotate protein interactions by other interaction databases (namely BIND [2], HPRD [17], MPACT [23] and GRID [24]). Note that this additional collection is a NOISY data set and thus not part of the ordinary TP collection, as these databases might have different annotation standards from MINT and IntAct (e.g. regarding the curation of genetic interactions). The test set is a balanced dataset, which contains 338 and 339 abstracts for TP and TN respectively.

We randomly selected 105,000 abstracts as our unlabeled dataset from the dataset used in the adhoc retrieval subtask of Genomic TREC 2004. It consisted of 10-year (from 1994 to 2003) published MEDLINE abstracts (4,591,008 records).

### Selecting likely-positive and negative instances

The limited training set contains only limited numbers of true-positive (TP) and true-negative (TN) data. To increase the generality of the classification model, we make use of the LP dataset from BioCreAtIvE-II IAS. However, most of the LP only annotate a selection of all the PPI types defined in Gene Ontology. This means that abstracts annotated in that database can only be treated as likely-positive examples, some of which may need to be filtered out. Another problem is that there are no negative data or even likely-negative data in any curation.

Liu et al. [25] provide a survey of these bootstrapping techniques, which iteratively tag unlabeled examples and add those with high confidence to the training set.

In the filtering process, two criteria must be considered: reliability and informativeness. We only retain sufficiently reliable instances, or the remainder will confuse the final model.

The informativeness of an instance is also important. We do not need additional instances if they are absolutely positive or negative. Deciding their labels is trivial for our initial classification model. In the terminology of SVM, they are not support vectors since they contribute nothing to the decision boundary in training. In testing, their output values by SVM are always greater than 1 or less than -1, which means they are distant from the separating hyperplane. Therefore, we can discard such uninformative instances to reduce the size of the training set without diminishing performance.

Following these criteria, we now illustrate our filtration process. The flowchart of the whole procedure is shown in Figure 2. We use the initial model trained with TP+TN to label the LP data we collected. Those abstracts in the original LP with an SVM output in [*γ*^+^, 1] are retained. The dataset after filtering out irrelevant instances in LP is referred to as 'selected likely-positive data' (LP*).

The construction of selected likely-negative (LN*) data is similar. We collect 50 k unlabeled abstracts from the PubMed biomedical literature database and classify them by our initial model. The articles with an SVM output in [-1, *γ*^-^] are collected into the LN* dataset.

The two thresholds *γ*^+ ^and *γ*^- ^are empirically determined to be 0 and -0.9, respectively. We use a looser threshold to filter LP data because of our prior knowledge of their reliability: after all, they have been recorded as PPI-relevant in some databases.

### Exploiting likely-positive and negative data

The final issue is how to utilize these filtered instances. Here we propose two different strategies. One is to incorporate LP* into TP and LN* into LN directly and use the expanded TP and TN to train a new classification model, called a mixed model. The other is use LP* and LN* to construct another model and incorporate its output into the underlying model. This is called a hierarchical model.

**Figure 7:**
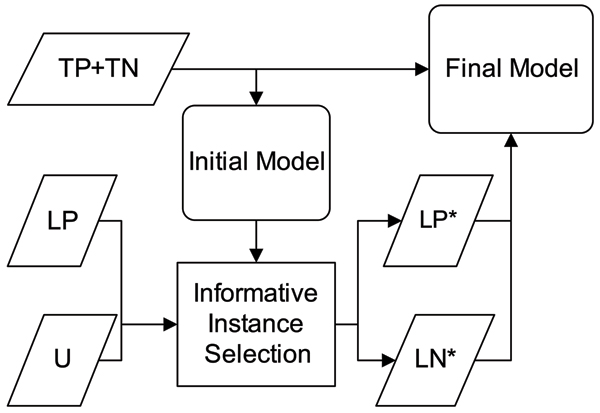
The flowchart of constructing the mixed model.

In the mixed model, as shown in Figure 7, the likely data are directly added back into the training set. This will enlarge the vocabulary and feature space, and thus increase the generality as long as the added data are reliable.

**Figure 8:**
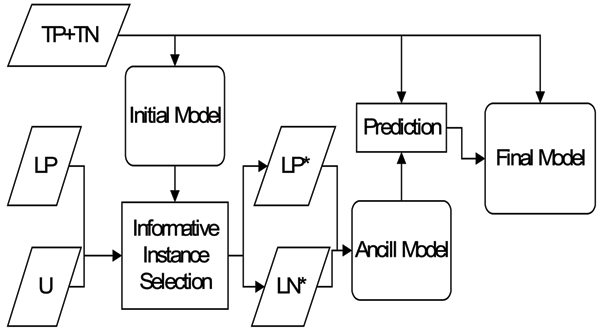
The flowchart of constructing the hierarchical model.

The hierarchical model is illustrated in Figure 8. The likely data (LP* + LN*) are used to train another SVM model, the ancillary model, which is completely independent of the original training set. Subsequently, we use the ancillary model to predict TP and TN instances, though their labels are already known, and these predicted values are scaled by a factor *κ *and encoded as additional features in the final model. In this manner, the final model can assign a suitable weight to the output of the ancillary model based on its accuracy in predicting the training set, which is assumed to be close to the accuracy in predicting the test set. The scaling factor *κ *can be regarded as a prior confidence in the ancillary model.

## Competing interests

The authors declare that they have no competing interests.

## Authors' contributions

RTHT designed all the experiments and wrote the paper with inputs from HJD and YWL. HCH wrote all programs, conducted all experiments, and wrote the Results and Discussion sections. WLH guided the whole project.
